# Suprapubic pressure: A marker of cervical insufficiency? A case report

**DOI:** 10.1016/j.eurox.2025.100411

**Published:** 2025-06-18

**Authors:** Athina Efthymiou, Effrosyni Birbas, Theofilos Kanavos, Charikleia Skentou, Nadia Almousa, George Makrydimas

**Affiliations:** Department of Obstetrics and Gynecology, School of Medicine, University of Ioannina, Ioannina, Greece; School of Medicine, University of Ioannina, Ioannina, Greece; Department of Obstetrics and Gynecology, School of Medicine, University of Ioannina, Ioannina, Greece

Dear Editor,

We report a case of a 33-year-old, low-risk woman with an uncomplicated pregnancy, who attended for her routine second trimester scan. She had a previous full term vaginal delivery and no risk factors for cervical insufficiency.

At 19^+5^ weeks of gestation a routine transvaginal cervical assessment was performed. Three measurements of the cervical length were obtained, over three minutes, and the shortest, 33.5 mm, was recorded.

However, in an attempt to better visualize the fetal brain transvaginally, mild pressure in the lower abdomen over the pubic symphysis was applied.

This pressure resulted in opening of the internal os and protrusion of the amniotic membranes into the cervical canal. Immediately, after relieving the pressure, the opening of the cervix disappeared. A few minutes later we repeated the suprapubic pressure with exactly the same effect (Video 1). Consequently, we applied fundal pressure for 15 s with no effect to the cervix. The woman did not experience any pain or contractions during the examination. In a repeat measurement of the cervix one hour later the cervical length was again 33 mm (Fig. 1, A-B).

**Fig. 1:**
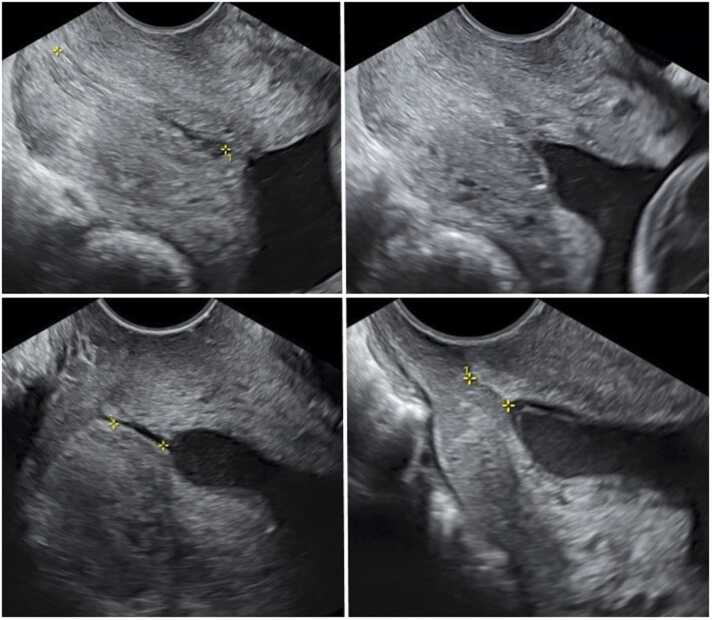
A (Upper left): Cervix before applying any pressure 19^+5^ weeks. Cervical length is 33.5 mm. B (Upper right): Cervix while applying suprapubic pressure. Note the opening of the internal os and protrusion of the amniotic membranes. C (Bottom left): Cervical length at 22^+6^ weeks measuring 10.1 mm with significant funneling. D (Bottom right): Cervical length at 23^+5^ weeks measuring 6.7 mm.

Supplementary material related to this article can be found online at: http://dx.doi.org/10.1016/j.eurox.2025.100411.

The following is the Supplementary material related to this article Video S1 and Video S2.

Video S1 Video Clip 1: Transvaginal Ultrasound of the cervix while applying suprapubic pressure. Note the opening of the internal os and protrusion of the amniotic membranes which disappeared shortly after. Supplementary material related to this article can be found online at

Video S2 Video Clip 2: Transvaginal Ultrasound of the cervix while applying suprapubic pressure in a different patient for comparison. In this case the internal os remains closed throughout. Supplementary material related to this article can be found online at

At the subsequent assessment at 22^+6^ weeks her cervix measured 10.1 mm and she was prescribed progesterone, vaginal pessaries 200 mg daily (Fig. 1, C). A week later, at 23^+5^ weeks, further shortening of the cervix to 6.7 mm occurred (Fig. 1, D) and cervical cerclage was performed according to our protocols [1].

At 37 weeks the cervical cerclage was removed and spontaneous onset of labour occurred a few hours later. She delivered vaginally the same day, a healthy female neonate of 2860gr.

## Discussion

Preterm birth remains the major problem in Obstetrics, Neonatology and Pediatrics. Screening with the measurement of the cervical length transvaginally, in the midtrimester, is the most successful method for the prediction of spontaneous preterm birth. However, only about 40 % of the women that will deliver before 34 weeks of gestation, have a short cervix at 20 weeks. Therefore, the majority of the pregnant women who will deliver preterm have a normal cervical length (>25 mm) in the midtrimester [2].

Previous studies have shown, that applying transfundal pressure may result in shortening of the endocervical canal along with an opening of the internal os and descent of the fetal membranes. [3]. In addition, they also reported that those women would benefit from a cervical cerclage [4]. However, although fundal pressure was described 30 years ago, there are no published data on the incidence and significance of this finding in low-risk populations in terms of risk for preterm delivery [3], [4], [5], [6].

To the best of our knowledge, this is the first report describing shortening of the cervix and opening of the internal cervical os following mild suprapubic pressure. Interestingly, this was apparently a woman with a very low risk for preterm birth as indicated not only by her cervical length that was well above the cut-off of 25 mm, which is considered short, but also by the fact that she had a previous delivery at term, which further reduces the risk in subsequent pregnancies. In addition, the application of fundal pressure had no effect to the cervix.

Opening of the internal os after applying suprapubic pressure can reveal a soft cervix which is unable to maintain its shape, irrespectively of the cervical length. Because of the closer proximity to the lower segment of the uterus, suprapubic pressure could be more effective compared to fundal pressure.

In that aspect, the application of supra pubic pressure, alone or in combination with fundal pressure, in women immediately after the routine cervical measurement, could detect cases with a normal cervical length but at high risk for cervical insufficiency and preterm birth.

## CRediT authorship contribution statement

**Skentou Charikleia:** Writing – review & editing. **Almousa Nadia:** Writing – review & editing, Visualization. **Makrydimas George:** Writing – review & editing, Supervision, Conceptualization. **Efthymiou Athina:** Writing – original draft, Data curation. **Birbas Effrosyni:** Writing – review & editing, Resources. **Kanavos Theofilos:** Writing – review & editing, Resources.

## Funding

The present study received no funding

## Declaration of Competing Interest

The authors have no conflicts of interest relevant to this article.
